# A High-Sensitivity, Bluetooth-Enabled PCB Biosensor for HER2 and CA15-3 Protein Detection in Saliva: A Rapid, Non-Invasive Approach to Breast Cancer Screening

**DOI:** 10.3390/bios15060386

**Published:** 2025-06-15

**Authors:** Hsiao-Hsuan Wan, Chao-Ching Chiang, Fan Ren, Cheng-Tse Tsai, Yu-Siang Chou, Chun-Wei Chiu, Yu-Te Liao, Dan Neal, Coy D. Heldermon, Mateus G. Rocha, Josephine F. Esquivel-Upshaw

**Affiliations:** 1 Department of Chemical Engineering, University of Florida, Gainesville, FL 32611, USA; cchiang@ufl.edu (C.-C.C.); fren@che.ufl.edu (F.R.); 2 Department of Electronics and Electrical Engineering, National Yang Ming Chiao Tung University, Hsinchu 30010, Taiwan; datsai1125.ee09@nycu.edu.tw (C.-T.T.); ericchou092.ee12@nycu.edu.tw (Y.-S.C.); williamchiu.ee10@nycu.edu.tw (C.-W.C.); yudoliao@nycu.edu.tw (Y.-T.L.); 3 Department of Surgery, University of Florida, Gainesville, FL 32611, USA; dneal@ufl.edu; 4 Department of Medicine, Division of Hematology and Oncology, University of Florida, Gainesville, FL 32611, USA; coy.heldermon@medicine.ufl.edu; 5 Department of Restorative Dental Science, Division of Prosthodontics, University of Florida, Gainesville, FL 32611, USA; mrocha@dental.ufl.edu (M.G.R.); jesquivel@dental.ufl.edu (J.F.E.-U.)

**Keywords:** breast cancer, biomarker, HER2, CA15-3, saliva, non-invasive, screening, printed circuit board, high sensitivity, biosensor

## Abstract

Breast cancer is a leading cause of cancer-related mortality worldwide, requiring efficient diagnostic tools for early detection and monitoring. Human epidermal growth factor receptor 2 (HER2) is a key biomarker for breast cancer classification, typically assessed using immunohistochemistry (IHC). However, IHC requires invasive biopsies and time-intensive laboratory procedures. In this study, we present a biosensor integrated with a reusable printed circuit board (PCB) and functionalized glucose test strips designed for rapid and non-invasive HER2 detection in saliva. The biosensor achieved a limit of detection of 10^−15^ g/mL, 4 to 5 orders of magnitude more sensitive than the enzyme-linked immunosorbent assay (ELISA), with a sensitivity of 95/dec and a response time of 1 s. In addition to HER2, the biosensor also detects cancer antigen 15-3 (CA15-3), another clinically relevant breast cancer biomarker. The CA15-3 test demonstrated an equally low limit of detection, 10^−15^ g/mL, and a higher sensitivity, 190/dec, further validated using human saliva samples. Clinical validation using 29 saliva samples confirmed our biosensor’s ability to distinguish between healthy, in situ breast cancer, and invasive breast cancer patients. The system, which integrates a Bluetooth Low-Energy (BLE) module, enables remote monitoring, reduces hospital visits, and enhances accessibility for point-of-care and mobile screening applications. This ultra-sensitive, rapid, and portable biosensor can serve as a promising alternative for breast cancer detection and monitoring, particularly in rural and underserved communities.

## 1. Introduction

Breast cancer remains a significant global health concern, with recent data indicating there is a substantial burden worldwide [1,2,3]. In 2022, there were approximately 2.3 million new cases of female breast cancer and 670,000 related deaths globally. Projections suggest that by 2050, annual cases could rise to 3.2 million, with 1.1 million deaths, disproportionately affecting countries with lower Human Development Index (HDI) scores [4]. In the United States, breast cancer continues to be the most commonly diagnosed cancer among women. In 2025, it is estimated that 316,950 new cases of invasive breast cancer will be diagnosed in women, along with 59,080 cases of ductal carcinoma in situ (DCIS). Additionally, approximately 42,170 women are expected to die from the disease [5]. These statistics highlight the critical need for effective diagnostic and monitoring strategies in order to manage and mitigate the impact of breast cancer.

Traditional diagnostic modalities, including mammography, ultrasound, and magnetic resonance imaging (MRI), remain the cornerstone for breast cancer detection. However, these techniques present limitations, such as high costs, limited accessibility, potential radiation exposure, and reduced sensitivity in detecting early-stage cancers, particularly in women with dense breast tissue [6,7,8,9,10]. To complement imaging, biomarker analysis has emerged as a pivotal tool in the diagnosis and management of breast cancer. Among biomarkers, human epidermal growth factor receptor 2 (HER2) is of particular interest. HER2 is a transmembrane receptor protein that, when overexpressed, is associated with aggressive tumor behavior and poorer prognosis [11,12,13,14,15,16]. Immunohistochemistry (IHC) is the standard clinical method for assessing HER2 expression in breast cancer diagnosis. This technique involves staining tissue biopsy samples with antibodies that bind to the HER2 protein, followed by microscopic evaluation to determine HER2 expression levels. The HER2 score is typically classified on a scale of 0 to 3+, where 0 and 1+ are considered HER2-negative, 2+ is equivocal and requires additional fluorescence in situ hybridization (FISH) testing, and 3+ is classified as HER2-positive, indicating eligibility for targeted HER2 therapies [17,18]. While IHC is widely used in clinical practice, it requires invasive biopsy collection, specialized laboratory equipment, and trained pathologists, leading to prolonged turnaround times [19]. In contrast, our biosensor provides a non-invasive, real-time, and highly sensitive alternative for HER2 detection in saliva, significantly reducing diagnostic delays and improving accessibility for early breast cancer screening.

Cancer antigen 15-3 (CA15-3) is a mucin-type glycoprotein whose levels are commonly elevated in breast cancer patients, and it has been extensively used as a circulating biomarker for monitoring disease progression and treatment response [20,21,22]. Recent studies have shown that CA15-3 is not only present in blood but can also be detected in saliva, offering a promising non-invasive alternative for biomarker screening [23,24]. The presence of CA15-3 in saliva is particularly advantageous for developing patient-friendly diagnostic tools, as it eliminates the need for blood to be drawn or invasive biopsies. This makes salivary CA15-3 a valuable target for point-of-care testing and remote monitoring applications, especially when combined with other breast cancer biomarkers such as HER2 to improve diagnostic sensitivity and specificity.

The enzyme-linked immunosorbent assay (ELISA) is commonly employed to quantify HER2 and CA15-3 levels in serum or saliva samples. While ELISA offers specificity, it requires trained personnel, involves multi-step protocols with prolonged processing times, and exhibits limited sensitivity. The detection limit for HER2 when using ELISA ranges from 10^−8^ to 10^−10^ g/mL, and CA15-3 detection generally falls within a similar range, typically around 10^−9^ g/mL [25,26]. These constraints underscore the need for more rapid, ultra-sensitive, and user-friendly diagnostic platforms that can enable accessible and real-time monitoring of breast cancer biomarkers.

In recent years, field-effect transistor (FET)-based biosensors have gained attention for their high sensitivity, label-free detection, and real-time monitoring capabilities. Various FET configurations, such as silicon nanowire FETs (SiNW-FETs) and graphene FETs (gFETs), have been explored for biosensing applications. However, traditional designs often require the entire sensor to be discarded after a single use, limiting practicality and increasing costs [27,28,29,30,31,32,33,34].

To address these challenges, we have developed an innovative biosensing platform that integrates a reusable PCB with disposable test strips for the detection of HER2 biomarkers. This system features a microprocessor unit (MCU) that controls various functional components, generates tunable test pulses, and digitizes the readout signals from the biosensor. A digital-to-analog converter (DAC) and level shifter provide adjustable voltage levels and pulse durations, optimizing detection conditions. The platform supports multi-channel measurements, enabling simultaneous detection of multiple biomarkers. The sensed signals are captured using a closed-loop amplifier with adjustable gain, enhancing linearity and signal fidelity. Unlike previous designs that required resetting to mitigate charge accumulation, our approach involves the use of synchronized pulse modulation to maintain measurement stability and accuracy.

One of the major advantages of this biosensor is its rapid detection speed, providing results in just one second, making this biosensor significantly faster than traditional diagnostic methods. Additionally, the integration of a Bluetooth Low-Energy (BLE) module enables wireless data transmission to a smartphone application, allowing healthcare professionals to monitor patients’ conditions remotely. This feature is particularly beneficial for reducing the need for frequent hospital visits, thereby improving patient convenience and reducing healthcare burdens.

The portability and ease of use of this device also make it highly suitable for screening in rural and underserved areas. This system can be deployed on mobile healthcare units, such as cancer-screening buses, to bring advanced screening capabilities to remote communities, ensuring broader access to early detection and timely intervention. In this study, we evaluated the performance of this novel biosensing platform by correlating the voltage output responses of the transistor with HER2 and CA15-3 concentrations in saliva samples collected from both healthy individuals and breast cancer patients. Our objective is to demonstrate the platform’s improved detection limits, enhanced sensitivity, and potential clinical applicability in breast cancer diagnostics.

## 2. Materials and Methods

**Figure 1:**
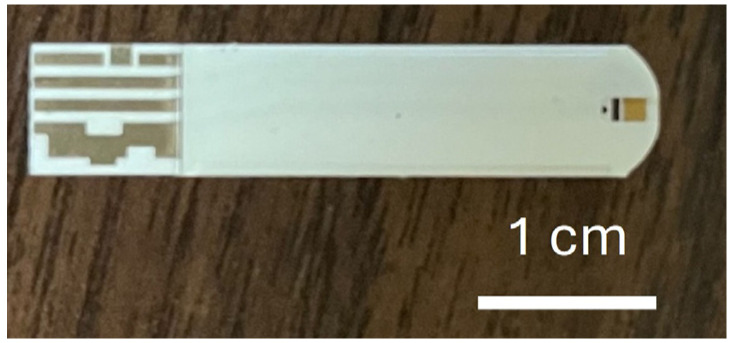
Photographic representation of the commercially available test strip used in this study, emphasizing the microfluidic channels and gold-plated electrode, which underwent functionalization for specific detection purposes.

In this study, commercially available glucose test strips (Luvnshare Biomedical Inc., Hsinchu, Taiwan) were utilized, as depicted in Figure 1. The strips were purchased without any glucose enzymes or pre-deposited chemicals, and only the bare structure—including the carbon electrode and microfluidic channel—was used. The strips are equipped with microfluidic channels at the tip for sample injection and incorporate a gold-plated electrode. The electrode underwent a series of functionalization steps with the HER2 and CA15-3 antibodies to enable the detection of sample variations. The functionalization process begins with ozone treatment for 15 min to remove carbon residues, followed by surface cleaning using a diluted ammonium hydroxide (NH_4_OH) solution to eliminate gold oxide. The channels are then rinsed with deionized (DI) water and dried with nitrogen.

Next, a 3-Mercaptopropanyl-N-hydroxysuccinimide ester (NHS ester) solution, prepared in ethanol, was applied [35,36]. NHS ester, featuring a three-carbon chain terminating in a thiol group and an N-hydroxysuccinimide ester, served as a bioconjugation agent, providing a reactive site for selective coupling with amine-containing molecules. The strips were immersed in this solution for 2 h, after which the channels were rinsed with DI water and dried with nitrogen. Subsequently, a HER2/ERBB2 monoclonal antibody (Sino Biological Inc., Chesterbrook, PA, USA) at a concentration of 20 μg/mL was introduced into the channels. For CA15-3 detection, an identical functionalization process was applied, using a monoclonal CA15-3 antibody (Sino Biological Inc., Chesterbrook, PA, USA) at the same concentration. The strips were then sealed and stored at 4 °C for 18 h. To deactivate any unfunctionalized groups and minimize potential interference, ethanolamine was applied. The successful functionalization of this antibody was confirmed in prior studies using standardized methods, including current–voltage and capacitance measurements. In addition, X-ray photoelectron spectroscopy (XPS) was used in prior work to confirm the presence of key chemical groups after each functionalization step [37]. Nanoparticle fluorescence imaging techniques have also been employed to visualize and verify surface coverage on a gold electrode [38]. In this study, strip quality was verified by screening with known concentrations of HER2 and CA15-3 proteins, and over 95% of the functionalized strips produced consistent digital output signals, confirming uniformity and high yield. For calibration, HER2 protein (Sino Biological Inc., Chesterbrook, PA, USA) was diluted to a series of concentrations using artificial saliva (Pickering Laboratories Inc., Mountain View, CA, USA) to establish a calibration curve. A parallel calibration curve for CA15-3 was established by diluting CA15-3 protein (Sino Biological Inc., Chesterbrook, PA, USA) in artificial saliva using the same procedure.

In addition to establishing the calibration curve using a series of diluted protein solutions, 29 human saliva samples were obtained from volunteers through the University of Florida Clinical and Translational Science Institute (UF CTSI) Biorepository. These samples included 11 samples from healthy volunteers, 5 from patients with in situ breast cancer, and 13 from patients with invasive breast cancer. The samples were collected from patients within the UF Health System and stored at −78 °C to preserve their integrity. All samples were de-identified and accompanied by corresponding clinical diagnoses, which were confirmed through biopsies as part of the patients’ routine care (UF IRB202101643). Upon being defrosted, the saliva samples were applied directly to the microfluidic channels without prior dilution, filtration, or centrifugation. Based on histological classification, the samples were categorized into three groups: (1) healthy controls, (2) in situ breast cancer, and (3) invasive breast cancer. Additionally, HER2 scores were available for 14 of the breast cancer samples, providing insight into HER2 concentration differences among these samples. The HER2 scores were derived from biopsy results using immunohistochemistry (IHC). All samples were tested using test strips functionalized with HER2 antibodies. The output digital readings were obtained by averaging ten consecutive pulse measurements, which were completed in approximately 1 s. Statistical analysis of the test results was performed using the Kruskal–Wallis test to determine *p*-values, ensuring robust evaluation of the data.

To further explore the diagnostic utility of salivary HER2 and CA15-3 levels, a support vector classification (SVC) model was employed to distinguish between healthy individuals and those requiring further breast cancer evaluation. The model used a polynomial kernel to capture nonlinear relationships between the two biomarkers, with a regularization parameter (C) of 0.1 and standard gamma scaling to balance feature influence. A binary classification approach was adopted by grouping in situ and invasive breast cancer cases into a single “requiring diagnosis” class. No class weighting was applied because of balanced performance regarding the natural class distribution. Cross-validation was performed to evaluate model robustness, and the final decision boundary was visualized using a 2D biomarker map (HER2 vs. CA15-3) for interpretability.

**Figure 2:**
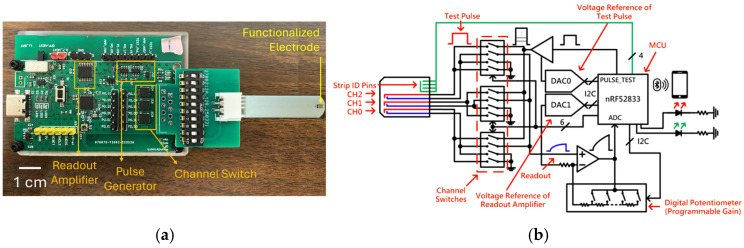
(**a**) Schematic representation of the printed circuit board (PCB) utilized in this study. (**b**) Functional block diagram illustrating the design architecture and components of the PCB.

Figure 2a displays a photograph of the proposed readout circuits, while Figure 2b shows a circuit diagram of this design. All components in the proposed module are commercially obtainable, facilitating swift mass production. The board features a microprocessor unit (MCU) responsible for controlling various functional parts, generating period-tunable pulses, and digitizing the readout signal from the biosensor. A level shifter and a digital-to-analog converter (DAC) adjust the voltage of the test pulses produced by the MCU, providing flexibility in both voltage and pulse periods based on experimental needs. The pulses then traverse switches to access specific channels. Importantly, the module allows for three-channel measurements, enabling simultaneous detection of three distinct biomarkers. After the pulses are applied to the sensor, a closed-loop amplifier captures the sensed signals. This closed-loop readout amplifier enhances linearity with adjustable gain tailored to the range of readout signals. The module has a pulsed signal with 1–3 V amplitude control and a pulse width range of 0.1 to 1.5 milliseconds. Data was acquired in an interval defined by the MCU to optimize the detection accuracy, resolution, and ranges of experimental results. Additionally, a Bluetooth LE module (nRF52833) was incorporated for wireless communication with a smartphone, which has an application that can control the adjustable parameters of the readout board. In addition, the onboard LEDs are indicators of battery charge level and debug functions. The adapter and cord for connecting the sensor strips were designed to heighten usability and availability. This design is notably smaller (WLH: 65 × 45 × 20 mm^3^) and more power efficient (~45 mW) compared to previous versions [15,39].

To operate the biosensor, the user begins by switching on the power using the onboard 3.7 V lithium battery connected to the PCB. Once powered, the biosensor pairs with a tablet via Bluetooth through a dedicated application. For testing, the patient’s saliva is first collected in a small sterile cup. The functionalized tip of the sensor strip is immersed in the saliva sample for approximately 3 s to ensure adequate antigen binding. After that, the strip is inserted into the strip connector located on the PCB, as shown in Figure 2a. The user then initiates the measurement by pressing the “Start” button in the mobile application, which immediately displays the digital output readings on the screen.

The protein concentration was simulated using commercial capacitors Integrated Into the PCB. To optimize the detection parameters, a series of characteristic tests were conducted on the PCB. These tests involved systematically varying the reference voltage, test voltage, and gain number settings to identify the optimal configuration for the target detection range.

## 3. Results and Discussion

**Figure 3:**
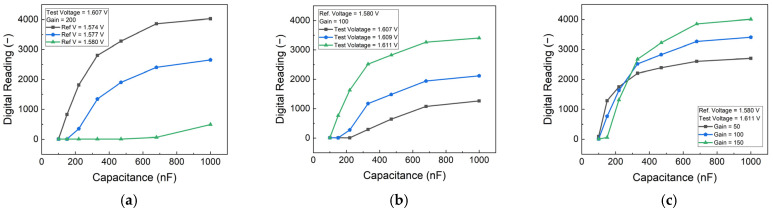
Impact of varying (**a**) reference voltage, (**b**) test voltage, and (**c**) gain settings on the performance of the PCB-based detection system.

The performance of the PCB-based HER2 detection system was optimized by analyzing the effects of reference voltage, test voltage, and gain settings. To simulate different HER2 concentrations, an external capacitor was employed, providing insights into the sensor’s response under controlled conditions. The results, as shown in Figure 3, indicate that increasing the difference between the test voltage and reference voltage enhances sensitivity. However, beyond a certain threshold, the sensor’s response begins to saturate, limiting its dynamic range. Therefore, an optimal voltage range was selected to maintain high sensitivity while preventing saturation. Figure 3c illustrates the impact of gain settings, showing that higher gain values improve sensitivity but also increase noise and signal variations. By carefully balancing these parameters, an appropriate gain setting was determined in order to achieve optimal performance with minimal noise interference. These adjustments ensured that the biosensor could operate within the desired detection range while maintaining accuracy and stability.

**Figure 4:**
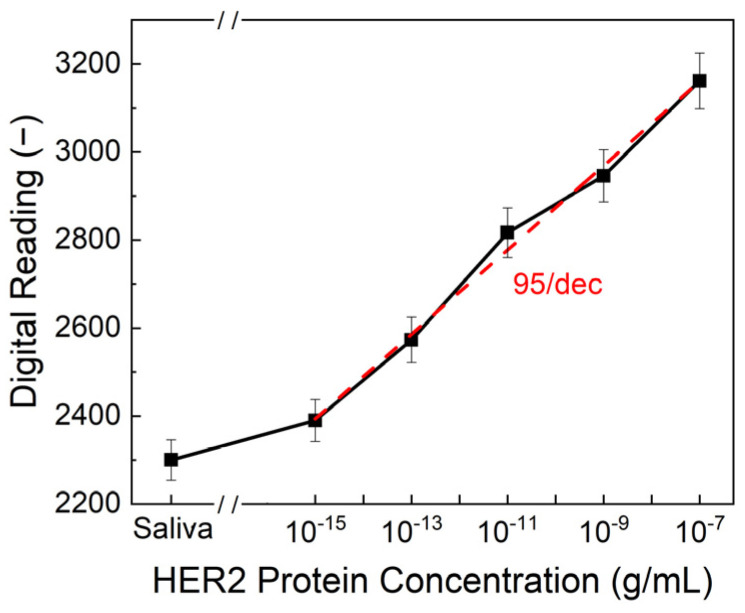
Output digital reading from PCB at different HER2 protein concentrations. The limit of detection is 10^−15^ g/mL, while the sensitivity is 95/dec.

To evaluate the sensitivity and reliability of the biosensor, HER2 protein solutions in both artificial saliva and standard buffer solutions were tested over a concentration range of 10^−7^ to 10^−15^ g/mL. The sensor demonstrated a strong correlation between HER2 concentration and the output digital readings from the PCB. As shown in Figure 4, the limit of detection (LOD) was determined to be 10^−15^ g/mL, with a sensitivity of 95/dec, meaning that for each order of magnitude increase in HER2 concentration, the output reading increased by approximately 95 units. This represents a significant advancement in comparison to conventional detection methods. ELISA, one of the most widely used techniques for HER2 quantification, typically achieves detection limits in the range of 10^−8^ to 10^−10^ g/mL, making our biosensor 4 to 5 orders of magnitude more sensitive. The ultralow detection limit of 10^−15^ g/mL was achieved through the use of double-pulse electrochemical measurements, which offer an improved signal-to-noise ratio compared to conventional single-pulse methods or ELISA. Additionally, while ELISA requires complex sample preparation, trained personnel, and prolonged processing times, our biosensor provides an ultra-fast response, delivering results in just one second. This rapid detection capability makes it ideal for real-time diagnostics and on-site testing.

**Figure 5:**
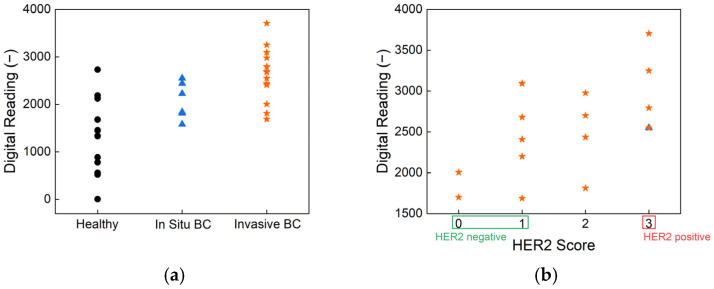
The output digital reading results from the human sample test with strips functionalized by HER2 antibody, classified by (**a**) breast cancer (BC) status and (**b**) HER2 score. The black dots are healthy volunteers, blue triangles mean In Situ breast cancer patients, and orange stars are invasive breast cancer patients.

To assess the clinical relevance of our biosensor, human saliva samples were tested using HER2-antibody-functionalized strips. The results, presented in Figure 5, show classification based on cancer status and HER2 score, demonstrating the effectiveness of the biosensor in distinguishing between different patient groups. Figure 5a illustrates the sensor’s ability to differentiate between healthy individuals, in situ breast cancer patients, and invasive breast cancer patients. The statistical analysis results confirm there are significant differences between these groups, highlighting the sensor’s potential for early-stage breast cancer detection. The overall *p*-value from the Kruskal–Wallis test is 0.001, indicating a statistically significant difference in biomarker readings across the three health statuses: healthy, in situ breast cancer, and invasive breast cancer. Pairwise comparisons further revealed a *p*-value of 0.0003 between healthy individuals and those with invasive breast cancer, demonstrating the biosensor’s strong ability to differentiate advanced disease from non-cancerous cases. The *p*-value between healthy and in situ breast cancer groups is 0.069 and that between in situ and invasive breast cancer is 0.084; these values approach statistical significance and suggest promising sensitivity for early-stage detection. These findings underscore the biosensor’s potential as a non-invasive, rapid, and reliable tool for stratifying disease stages based on salivary biomarker levels. Such performance, achieved without the need for invasive sampling or complex instrumentation, supports its application in clinical screening and monitoring—particularly in settings where conventional diagnostics are less accessible. Further, Figure 5b presents the classification of samples according to HER2 scores, showing a clear distinction between HER2-negative and HER2-positive cases. This performance aligns well with the current clinical standard, immunohistochemistry (IHC), which is widely used for HER2 evaluation. However, unlike IHC, which requires biopsy samples and processing times ranging from several hours to days, our biosensor provides a non-invasive alternative with real-time results, offering significant advantages in terms of speed and ease of use.

**Figure 6:**
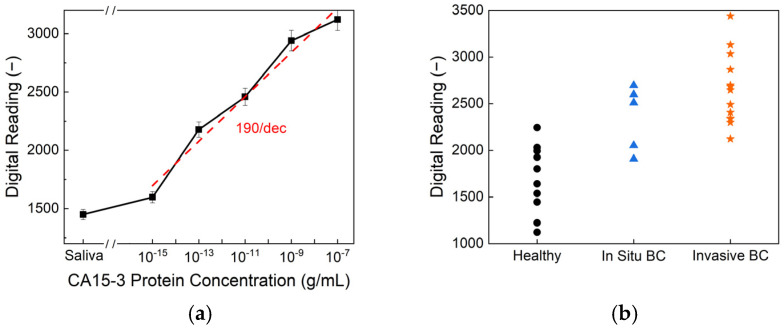
(**a**) Digital output readings of the biosensor under varying CA15-3 protein concentrations, demonstrating its limit of detection and sensitivity. (**b**) Biosensor responses from human saliva samples, classified according to health status: healthy, in situ breast cancer, and invasive breast cancer.

Figure 6 presents the CA15-3 detection performance of the biosensor. As shown in Figure 6a, the sensor exhibits a clear, concentration-dependent response across a wide dynamic range of CA15-3 protein concentrations, achieving an exceptional limit of detection of 10^−15^ g/mL and a high sensitivity of 190/dec. This level of sensitivity surpasses that of commercial ELISA kits, which typically exhibit detection limits of around 10^−9^ g/mL, highlighting the superior analytical performance of the proposed platform. The higher sensitivity observed for CA15-3 compared to HER2 can be attributed to the molecular properties of the biomarkers. CA15-3, a mucin-type glycoprotein, has a larger molecular size and a greater number of glycosylated epitopes, facilitating more extensive antibody binding and generating a stronger electrochemical response. In contrast, soluble HER2 has a smaller structure with fewer accessible epitopes, which may result in lower signal intensity and sensitivity during detection. Figure 6b displays the biosensor readings from human saliva samples, categorized by health status—healthy, in situ breast cancer, and invasive breast cancer. Statistical analysis reveals an overall *p*-value of <0.001, indicating a significant difference among the three groups. Specifically, the comparison between the healthy and in situ breast cancer samples yielded a *p*-value of 0.009, while the healthy-versus-invasive group showed even greater significance, with *p* < 0.0001. Although the in situ-versus-invasive comparison resulted in a *p*-value of 0.246, suggesting there is no significant difference between these two stages, the sensor effectively differentiated cancer patients from healthy individuals. These findings demonstrate the potential of CA15-3 as a viable salivary biomarker and validate the biosensor’s clinical utility for non-invasive breast cancer detection and monitoring.

**Figure 7:**
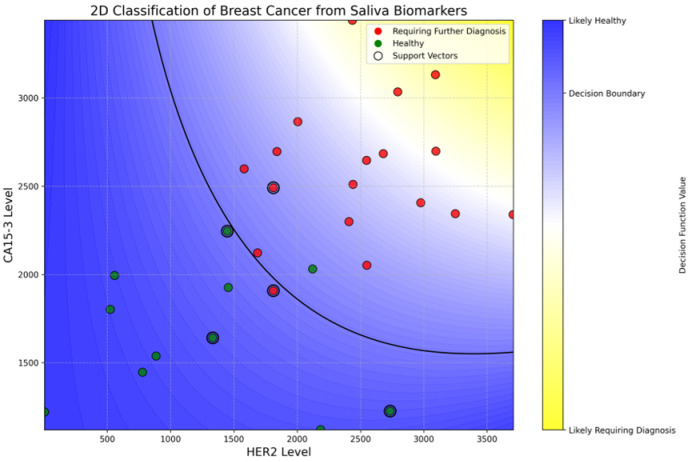
Two-dimensional classification of breast cancer risk based on salivary HER2 and CA15-3 levels using a support vector classification (SVC) model. The background color map represents the model’s decision function, where blue indicates regions classified as likely healthy and yellow indicates regions classified as likely requiring further diagnosis. The solid black curve denotes the decision boundary. Green dots represent healthy individuals, red dots represent individuals requiring further diagnosis (in situ or invasive breast cancer), and black-circled points are support vectors that define the classification boundary. This visualization demonstrates the model’s ability to distinguish between classes using salivary biomarkers with high accuracy and interpretability.

The SVC model achieved a promising test accuracy of 88.9% and an ROC AUC of 95%, demonstrating strong potential for non-invasive breast cancer screening using salivary biomarkers. As shown in Figure 7, the model successfully separated healthy individuals (green) from those requiring further diagnosis (red) based on HER2 and CA15-3 levels. Notably, the model achieved 100% recall for patients in the diagnostic group, ensuring that no cancer cases were missed, while its precision remained at 86%, reflecting a cautious classification strategy. The curved decision boundary suggests a synergistic interaction between HER2 and CA15-3, where elevated levels of either—or both—biomarkers increase cancer suspicion. A complete description of the training pipeline, hyper-parameter search, cross-validation metrics, confusion matrices, and the annotated Jupyter notebook is provided in Supplementary Information S1. These results reveal the value of using combined biomarker profiling in machine learning frameworks for early, accessible, and accurate breast cancer risk stratification.

The integration of Bluetooth Low-Energy (BLE) communication in our system enables remote monitoring of both HER2 and CA15-3 levels, allowing healthcare providers to track patient conditions without the need for frequent hospital visits. This feature is particularly beneficial for patients undergoing breast cancer treatment, as it facilitates continuous monitoring with minimal clinical intervention. Furthermore, the portability and user-friendly nature of this biosensor make it highly suitable for screening in rural and underserved areas where access to specialized medical facilities is limited. The device can be deployed in mobile cancer-screening units, such as healthcare buses, to provide point-of-care diagnostics and enhance early detection efforts in remote communities.

The findings from this study highlight the capacity of our biosensor to serve as a highly sensitive, rapid, and non-invasive diagnostic tool for salivary HER2 and CA15-3 detection. By achieving a detection limit of 10^−15^ g/mL and a response time of just one second, this technology outperforms existing methods, offering a practical solution for both clinical and point-of-care applications. The high sensitivity of 190/dec for CA15-3 and 95/dec for HER2 further demonstrates this system’s strong analytical performance. Furthermore, its ability to integrate wireless data transmission and remote monitoring expands its utility beyond traditional diagnostic settings, enabling continuous tracking of multiple breast cancer biomarkers without the need for frequent hospital visits. The system’s adaptability for use in mobile screening programs also provides a scalable solution for improving early breast cancer detection, particularly in underserved regions. Although reproducibility and sensitivity have been demonstrated using real clinical saliva samples, the current study should still be regarded as a proof of concept, with broader validation efforts needed to support clinical adoption. Overall, the combination of ultra-low detection limits, real-time responses, multi-biomarker capability, and remote monitoring positions this biosensor as a transformative technology in breast cancer diagnostics and management.

## 4. Conclusions

This study presents the development and validation of a highly sensitive, rapid, and non-invasive biosensor for breast cancer detection, capable of simultaneously detecting HER2 and CA15-3 biomarkers in saliva. By integrating a reusable PCB with functionalized glucose test strips, the system achieved a remarkable limit of detection of 10^−15^ g/mL for both biomarkers, surpassing conventional ELISA methods by 4 to 5 orders of magnitude. The biosensor demonstrated sensitivities of 95/dec for HER2 and 190/dec for CA15-3, with a rapid response time of just 1 s, offering a significant advantage over traditional diagnostic techniques. Clinical validation using saliva samples confirmed the biosensor’s effectiveness in distinguishing between healthy individuals, in situ breast cancer patients, and invasive breast cancer patients, supporting its potential for real-world applications. The CA15-3 results, in particular, showed strong statistical significance between healthy and cancer groups (overall *p* < 0.001), reinforcing its clinical relevance as a complementary biomarker. Additionally, the integration of Bluetooth Low-Energy (BLE) communication enables remote patient monitoring, reducing the need for frequent hospital visits and facilitating continuous tracking of biomarker levels. The device’s portability and ease of use make it particularly well-suited for point-of-care applications and mobile cancer-screening programs, enhancing the availability of early breast cancer detection, particularly in rural and underserved areas. The combination of ultra-high sensitivity, a rapid response time, dual-biomarker capability, and remote monitoring positions this biosensor as a promising proof-of-concept tool for breast cancer screening and disease management. Future work will focus on expanding this biosensor’s clinical validation and optimizing multi-biomarker integration to further enhance its diagnostic accuracy and broaden its applications in personalized medicine.

## 5. Patents

Biosensor Detection of Breast Cancer Staging in Saliva Biomarkers (PCT/US2024/60095) (provisional patent).

## Data Availability

The data that support the findings of this study are available upon request.

## Supplementary Materials

The following supporting information can be downloaded at https://www.mdpi.com/article/10.3390/bios15060386/s1.

## Abbreviations

The following abbreviations are used in this manuscript:

HER2	Human Epidermal Growth Factor Receptor 2
CA15-3	Cancer Antigen 15-3
IHC	Immunohistochemistry
PCB	Printed Circuit Board
BLE	Bluetooth Low Energy
HDI	Human Development Index
DCIS	Ductal Carcinoma In Situ
MRI	Magnetic Resonance Imaging
FISH	Fluorescence In Situ Hybridization
ELISA	Enzyme-linked Immunosorbent Assay
FET	Field-Effect Transistor
SiNW-FET	Silicon Nanowire Field-Effect Transistor
gFET	Graphene Field-Effect Transistor
MCU	Microprocessor Unit
DAC	Digital-to-Analog Converter
LOD	Limit of Detection
