# Supplementary material for: A High-Sensitivity, Bluetooth-Enabled PCB Biosensor for HER2 and CA15-3 Protein Detection in Saliva: A Rapid, Non-Invasive Approach to Breast Cancer Screening
Source: Biosensors (Basel). 2025 Jun 15;15(6):386. doi: 10.3390/bios15060386 (PMC12190843; doi:10.3390/bios15060386)
Supplement: Supplementary file 1 [file biosensors-15-00386-s001.zip › biosensors-3637868-supplementary.pdf]

Supplementary 1 - Machine Learning Analysis of Salivary Biomarkers

Data Preprocessing and Feature Engineering

We analyzed a dataset consisting of 29 saliva samples containing levels of two breast cancer biomarkers (HER2 and CA15-3) along with clinical status (healthy, in situ carcinoma, or invasive cancer). To prepare the data for machine learning, we first cleaned and transformed the raw features. Both biomarker distributions were right-skewed with values ranging from single digits up to several thousands, so a log10-transformation was applied to the HER2 and CA15-3 measurements to reduce skewness and stabilize variance. After log transformation, we standardized the features using z-score scaling (zero mean, unit variance) via StandardScaler to ensure both biomarkers had comparable scales. This scaling is important for algorithms like support vector machines as it ensures that one feature does not dominate due to a larger numeric range. All samples had complete data for the two biomarkers, so no imputation was required (i.e., there were no missing values). The outcome variable was initially multi-class (1 = healthy, 2 = in situ, and 3 = invasive); however, for the purpose of a binary classification (screening healthy vs. cancerous patients), we binarized the labels such that healthy = 0 and cancer (either in situ or invasive) = 1. This framing aligns with the screening scenario, where we distinguish healthy individuals from those who may require further diagnostic workup. In summary, the preprocessing pipeline included log-transforming HER2 and CA15-3, feature scaling, and defining a binary target for “cancer detection” (the presence vs. absence of disease).

Model Selection and Justification

Given the small sample size (N=29) and since there were only two features, we considered a range of classification algorithms known to perform well in low-data regimes. In our preliminary experiments, we evaluated various classifiers (logistic regression, decision trees, naive Bayes variants, neural networks, etc.), and we observed that support vector machines (SVMs) tended to perform best in separating the classes. We ultimately selected an SVC (support vector classifier) as our modeling approach due to SVMs’ effectiveness in high-dimensional or limited-sample settings and their ability to capture non-linear decision boundaries through kernel functions. In particular, the polynomial kernel SVC was chosen based on its superior performance during model selection. The polynomial kernel yielded a curved decision boundary in the HER2–CA15-3 feature space, which intuitively fit the data better than a linear separator. This suggests a synergistic or interaction effect between the two biomarkers—i.e., certain nonlinear combinations of HER2 and CA15-3 levels were indicative of cancer status, which a polynomial kernel could capture. By contrast, a linear kernel was too restrictive (misclassifying more cases), and an RBF (Gaussian) kernel, while flexible, did not outperform the polynomial in cross-validation (and risked overfitting given the very small dataset). The chosen polynomial SVC model also provides a geometrically interpretable decision boundary (a conic section curve in 2D) that aligns with domain intuition: elevated levels of either or both biomarkers push a sample

into the “cancer” region of the feature space. Additionally, SVMs inherently focus on support vectors (critical samples near class boundaries), an advantageous factor in this case in regard to generalizing from only a handful of examples. In summary, the use of an SVC with a polynomial kernel was justified both by its empirical performance and the need for a non-linear yet interpretable classifier to capture interactions between HER2 and CA15-3.

Hyperparameter Tuning and Cross-Validation

To optimize the SVC model, we conducted an extensive hyperparameter search using grid search with cross-validation. The following SVC hyperparameters were tuned: the regularization parameter C (tested values 0.1, 1, 10, and 100), the kernel function (evaluating 'linear', 'poly', 'rbf', and 'sigmoid' kernels), the kernel coefficient gamma (for non-linear kernels, tested 'scale' (default inverse variance), 'auto', and the fixed values 0.01 and 0.001), and the class weighting (either standard unweighted or 'balanced' to compensate for class imbalance). This grid comprised 4×4×4×2 = 128 combinations, which were each evaluated via cross-validation. We applied a 5-fold-stratified cross-validation strategy (StratifiedKFold (n_splits = 5, shuffle = true, random_state = 42)) to the training data to ensure each fold maintained the same ratio of healthy to cancerous samples. Given the moderate class imbalance (38% healthy vs. 62% cancerous), model selection was driven by the F1-score as the scoring metric in GridSearchCV. Optimizing for F1 (the harmonic mean of precision and recall) helped balance sensitivity and precision, an important facet in a diagnostic context (we desired to both catch as many cancers as possible and minimize false alarms). The grid search procedure trained and evaluated each candidate model across the folds and identified the combination yielding the highest mean F1. The grid search results revealed that the best SVC model had the following parameters: C = 0.1, kernel = ‘poly’ (polynomial), gamma = ‘scale’, and class_weight = none (no special weighting). Notably, this best model used the polynomial kernel (confirming our choice of model) and a relatively small C (0.1), indicating that a simpler, more regularized model generalized best (likely to avoid overfitting in this small dataset). The fact that the optimal gamma is ‘scale’ means the polynomial kernel’s coefficient was effectively set to a value adapting to the data’s variance. With these hyperparameters set, we retrained the SVC on the full training set and then assessed its performance. For a robust estimate of generalization performance, we also performed a fresh 5-fold-stratified cross-validation on the entire dataset using the best model (this yielded performance metrics with all 29 samples participating in folds, allowing us to leverage all the data in evaluating variability).

Model Performance Evaluation

Cross-Validation Performance: Over the 5-fold-stratified CV (using the best SVC model), the classifier showed strong and balanced performance. The mean accuracy was ~0.8333 (83.3%), with a standard deviation of ± 0.1826 across folds, indicating some variability likely due to the low sample count per fold. The precision (for the positive class = cancer) averaged 0.8000 (80.0% ± 26.7%), and recall averaged 0.8000 (80.0% ± 24.5%). The mean F1-score was 0.7733

(77.3% ± 22.6%). Importantly, the mean ROC AUC (area under the receiver operating characteristic curve) was 0.9500 (95.0% ± 10.0%), reflecting an excellent ability to rank/order healthy vs. cancer samples (despite one fold likely achieving an AUC of 1.0 and another fold achieving an even lower value, hence the ±0.10 deviation). These cross-validation results suggest the model can generalize reasonably well, though the relatively high standard deviations underscore the uncertainty due to the limited data. Nonetheless, an AUC near 0.95 in CV indicates a model's underlying ranking of risk is highly accurate, which is critical for a screening tool (meaning we can set a threshold to achieve high sensitivity if desired). Test Set Performance: Finally, we evaluated the tuned model on an independent test set comprising 30% of the data (nine samples that were not included in training). The model achieved 88.9% accuracy on the test set (with eight out of nine samples correctly classified). The single misclassification was a healthy sample that was predicted to be "cancer risk," reflecting an intentional bias of the model towards erring on the side of caution. The confusion matrix of the test results is shown below.

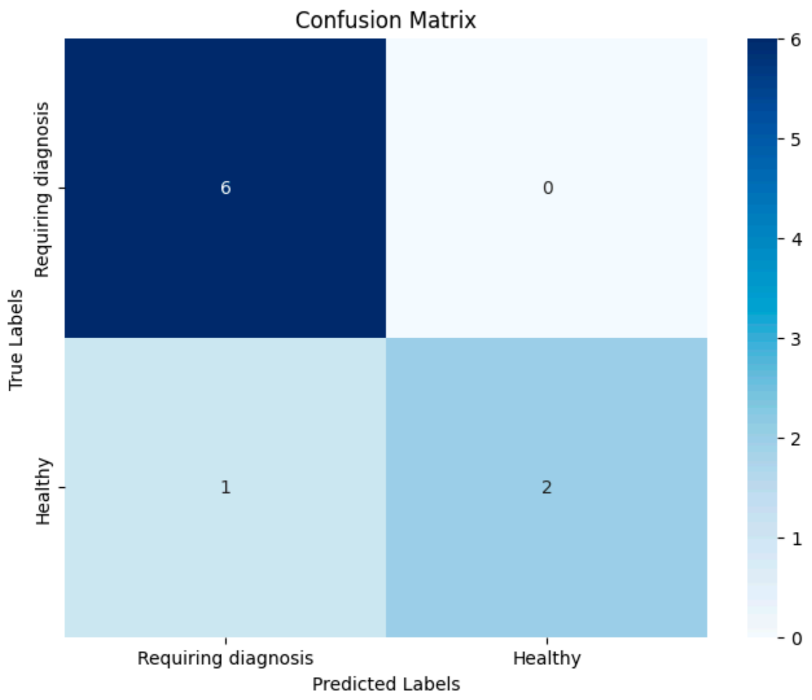

Supplementary Figure 1: Confusion matrix of the SVC predictions on the test set. Rows represent true class (Cancer vs. Healthy) and columns represent predicted class. The model correctly identified all six patients with in situ or invasive cancer as needing further diagnosis (with no false negatives for cancer) and correctly identified two out of three healthy individuals. One healthy sample was incorrectly flagged as cancerous (false positive). This outcome

corresponds to a 100% sensitivity (recall) for detecting cancer cases, since all the actual cancer patients were caught by the model, and a 66.7% specificity for ruling out disease (only 2/3 of the healthy cases were correctly classified as healthy). In other terms, for the cancer (positive) class, the model's precision was 85.7% (6/7 of those it labeled as "cancer" truly had cancer), and recall was 100% (with 6/6 cancer cases detected). For the healthy (negative) class, precision was 100% (the model did not label any cancer patient as healthy, so every patient predicted to be healthy was truly healthy), and recall was 66.7% (only two of the three actual healthy cases were predicted to be healthy). The F1-score for the healthy class was 0.80, and that for the cancer class was 0.92, with a macro-average F1 of ~0.86. These metrics illustrate the model's deliberate tuning for high sensitivity: it prefers to cast a wider net for potential cancers, even if it means one healthy individual is falsely flagged (and this individual would then undergo additional screening to confirm whether they are cancer-free).

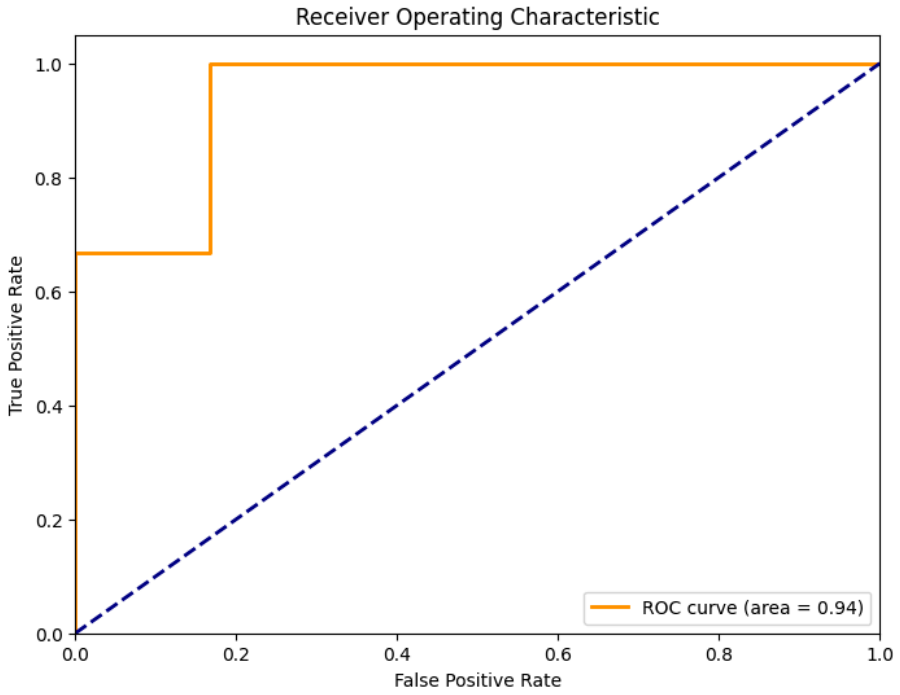

Supplementary Figure 2: Receiver operating characteristic (ROC) curve for the SVC on the test set (using cancer as the positive class). The orange curve shows the true-positive rate (sensitivity) vs. false-positive rate for varying classification thresholds, and the dashed blue line is the no-discrimination baseline (AUC = 0.50). The area under the ROC curve (AUC) is approximately 0.94 for this test, indicating outstanding discriminative ability. In fact, the curve rises quickly toward the top left, reflecting that the model can achieve very high sensitivity with only a minor increase in the false-positive rate. In our case, we were able to choose a threshold that yielded perfect sensitivity with only ~33% false positives (as observed). The high AUC confirms that the model's ranking of samples by predicted risk is nearly ideal—all actual cancer cases received higher risk scores than the healthy cases (aside from one healthy case with an intermediate

score), mirroring the confusion matrix findings. Overall, the test set evaluation demonstrates that the SVC model, with proper tuning, successfully separates healthy vs. cancerous cases using salivary HER2 and CA15-3 levels, achieving nearly 89% accuracy on this small independent set. Crucially, it did not miss any cancer cases (i.e., there were no false negatives), which is paramount in a screening context, and it kept the false-positive rate at an acceptable level for follow-up diagnostics.

Software and Libraries Used
